# Cardiotoxic effects of pantoprazole in Wistar rats submitted to cardiac ischemia and reperfusion

**DOI:** 10.1590/1806-9282.20251415

**Published:** 2025-12-15

**Authors:** Adilson Costa dos Santos, Celia Maria Camelo Silva, Erisvaldo Amarante de Araújo, Lucas de Oliveira Sassi, Adriano Caixeta, Murched Omar Taha, Marcelo Pires-Oliveira, Afonso Caricati-Neto, Renato Delascio Lopes, Fernando Sabia Tallo, Francisco Sandro Menezes-Rodrigues

**Affiliations:** 1 Universidade Federal de São Paulo – São Paulo (SP), Brazil.; 2 Centro universitário UNIME – Lauro de Freitas (BA), Brazil.; 3 Associação Médica Brasileira – São Paulo (SP), Brazil.; 4 Duke University – Durham (NC), United States.

**Keywords:** Cardiac arrhythmias, Pantoprazole, Myocardial ischemia, Myocardial reperfusion, Myocardial reperfusion injury, Cardiac arrhythmia

## Abstract

**OBJECTIVE:**

The aim of the study was to evaluate the cardiotoxic effects of pantoprazole in Wistar rats submitted to cardiac ischemia and reperfusion.

**METHODS:**

Cardiac ischemia and reperfusion was induced in rats through tourniquet of the left anterior descending coronary artery (10 min) followed by reperfusion (75 min). The incidences of ventricular arrhythmias, atrioventricular block, and lethality were evaluated by electrocardiogram analysis and compared between the control group (saline solution+cardiac ischemia and reperfusion) and the animals treated with pantoprazole 36 mg/kg (pantoprazole+cardiac ischemia and reperfusion), intravenously, immediately before cardiac ischemia (pantoprazole+cardiac ischemia and reperfusion) or before cardiac reperfusion (pantoprazole+cardiac reperfusion).

**RESULTS:**

There were no differences between the incidences of ventricular arrhythmias in the groups studied. Treatment with pantoprazole before cardiac ischemia and reperfusion increased the incidence of atrioventricular block and lethality and myocardial injuries compared to the saline solution+cardiac ischemia and reperfusion group, but treatment of animals with pantoprazole before cardiac reperfusion decreased these parameters and serum creatine kinase-MB levels.

**CONCLUSION:**

Our results indicate that when pantoprazole is administered before cardiac ischemia and reperfusion, it promotes cardiotoxicity; however, when administered before cardiac reperfusion, it promotes cardioprotection in Wistar rats.

## INTRODUCTION

Cardiovascular diseases (CVDs) are among the leading causes of death worldwide, both in economically wealthy and developing countries. Among the CVDs with the greatest impact on patient mortality and morbidity are arterial hypertension, acute coronary syndrome, ischemic heart disease, and acute myocardial infarction (AMI)^1,2^. In AMI, there is a significant reduction in the supply of oxygen, glucose, and other nutrients necessary for maintaining myocardial bioenergetics and physiology, which seriously impairs cardiac contraction capacity and, consequently, cardiac contractility, leading to the emergence of serious and potentially fatal cardiac arrhythmias^3-5^. CVDs are a cause for public health concern worldwide, as they are one of the leading causes of morbidity and mortality.

Several studies have shown that the use of proton pump inhibitors (PPIs), used to treat various diseases such as dyspepsia, gastroesophageal reflux disease, and Barrett's esophagus due to the fact that they block the production of gastric acid in the stomach, can increase the incidence of major adverse cardiovascular events (MACE)^6-9^. Among the mechanisms of PPIs related to CVD is the inhibition of the enzyme dimethylarginine dimethylaminohydrolase (DDAH), the enzyme responsible for the degradation of asymmetric dimethylarginine (ADMA), an endogenous inhibitor of the nitric oxide synthase (NOS) that produces nitric oxide (NO)^10-18^.

Increased plasma ADMA levels are associated with an increased risk of CVD due to increased vascular inflammation, thrombosis, and elevated endothelial oxidative stress^10-18^. Therefore, we decided to evaluate the cardiotoxic effects of pantoprazole in Wistar rats submitted to CIR.

## METHODS

### Animals

Adult male Wistar rats weighing 290–320 g were maintained at 21±2°C with a 12:12-h light/dark cycle and were given food and water ad libitum. This study was approved by the Ethics Committee of the Escola Paulista de Medicina—Universidade Federal de São Paulo (9728120921, approved in the meeting of 10/14/2021). The rats were randomly allocated to the following groups:

1. SHAM group (n=10): sham-operated group;
2. SS+CIR group (n=14): received saline solution (SS) intravenously before Ischemia;
3. PANT+CIR group (n=14): received 36 mg/kg of pantoprazole (PANT), intravenously, before cardiac Ischemia;
4. PANT+CR group (n=14): received 36 mg/kg of PANT intravenously after cardiac Ischemia but before cardiac reperfusion.

All animals underwent a 15-min stabilization period, 10 min of cardiac Ischemia, and 75 min of cardiac reperfusion. All experiments were monitored by electrocardiogram throughout the experimental protocol.

### Biochemical evaluation of serum markers of cardiac injury

Blood samples were extracted from the abdominal aorta at the end of the reperfusion period, centrifuged for 40 min at 2,500 rpm, 5°C, and stored at -20°C. Using a commercial kit (Katal Biotecnológica Ind. Com. Ltda., Belo Horizonte, MG, Brazil) and a kinetic ultraviolet approach with a measuring point of 340 nm, creatine kinase MB fraction (CK-MB) was quantitatively determined^19^.

### Histopathological analysis of left ventricular myocardial tissue

Cardiac left ventricle fragments were divided into three portions (apex, mid, and distal regions) with roughly comparable thickness using axial cross sections. Mid sections were cross-sectioned (4–5 μm width) and stained with hematoxylin-eosin (H&E). A qualified pathologist blindly assessed the slides using light microscopy (400× and 1,000×) for lesions associated with CIR: presence of hyperemic blood vessels, pyknosis, inflammatory infiltration, cardiomyocyte degeneration, loss of striation, and interstitial edema^19^.

### Statistical analysis

Fisher's exact test was used to compare the percentage-based ventricular arrhythmias (VA), atrioventricular block (AVB), and lethality (LET) incidences. Tukey's post hoc test was used to compare the mean±standard error of the mean (SEM) of CK-MB levels using a one-way analysis of variance. For statistical analysis, GraphPad Prism 8.0 (GraphPad Software Inc., La Jolla, CA, USA) was utilized^19^.

## RESULTS

Impact of pantoprazole on the frequency of ventricular arrhythmias, atrioventricular block, and lethality

**Figure 1:**
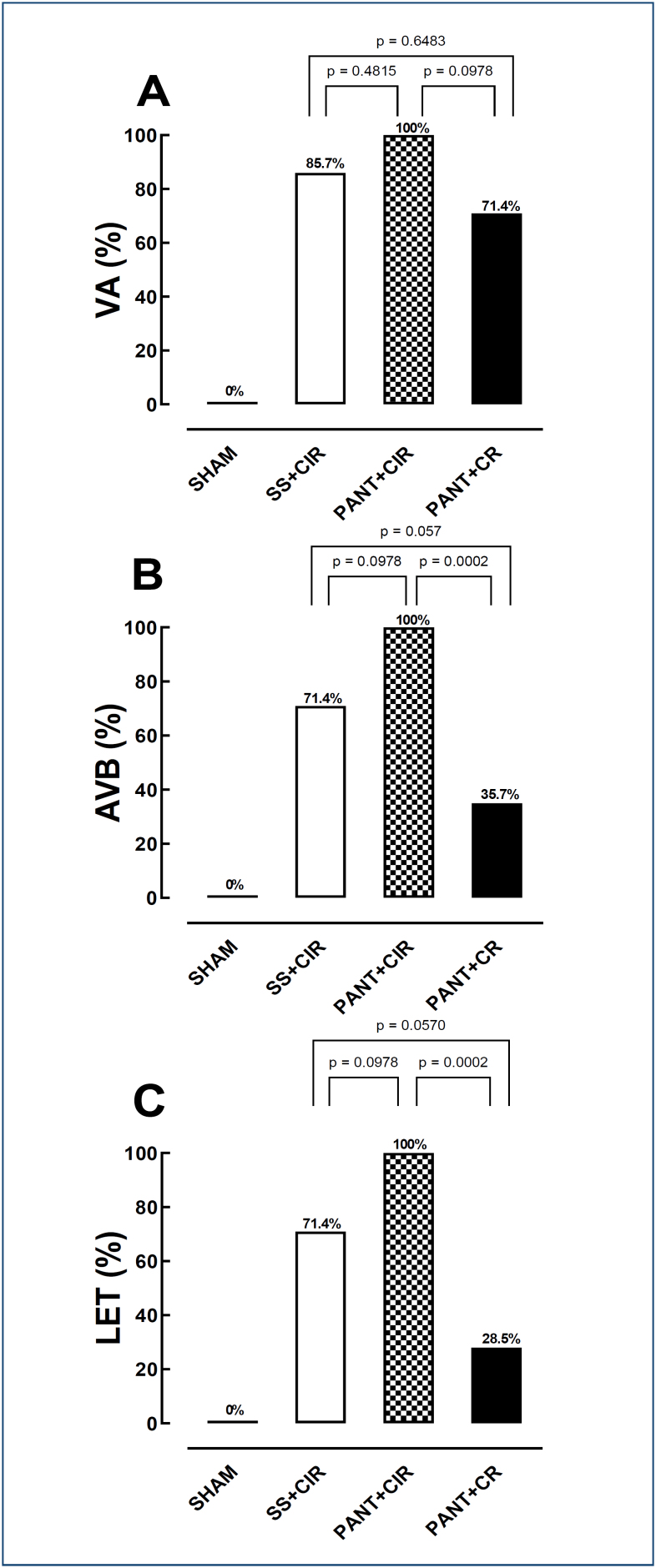
Histograms representing the (A) incidence of ventricular arrhythmias, (B) atrioventricular block, and (C) lethality in the SHAM, saline solution+cardiac ischemia and reperfusion, pantoprazole+cardiac ischemia and reperfusion, and pantoprazole+cardiac reperfusion groups. Fisher's exact test was used to compare the percentage-based ventricular arrhythmias, atrioventricular block, and lethality incidences.

There was no difference in the incidence of VA between the different groups. However, important differences were observed in the incidence of AVB and LET between the groups analyzed, as treatment with PANT before CIR increased the incidence of both AVB and LET, while administration of PANT before RC decreased it (Figure 1).

### Impact of pantoprazole on the serum concentration of creatine kinase-MB

**Figure 2:**
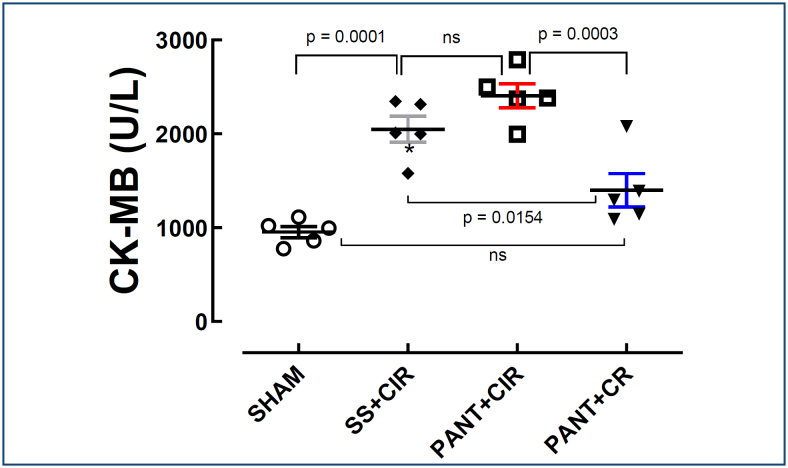
Serum creatine kinase-MB concentrations of the SHAM, pantoprazole+cardiac ischemia and reperfusion, and pantoprazole+cardiac reperfusion groups. The results were expressed as mean±standard error of the mean, and analysis of variance was applied, followed by Tukey's post-test.

There was a difference in the serum concentrations of CK-MB in the SHAM (952.8±59.8 U/L), SS+CIR (2,050±138.3 U/L), PAN+CIR (2,407±127.8 U/L), and PAN+CR (1,400±177.9 U/L) groups, as treatment with PANT before CIR increased the concentration of CK-MB while administration of PANT before CR decreased it (Figure 2).

### Impact of pantoprazole on myocardial injuries

**Figure 3:**
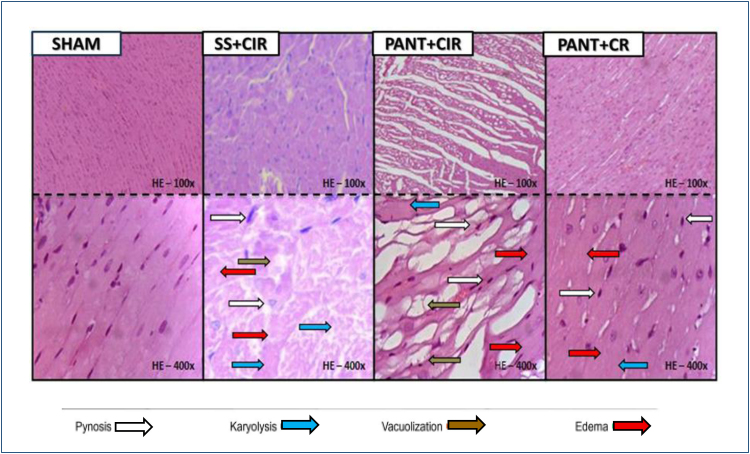
Photomicrographs of the myocardium of the SHAM, saline solution+cardiac ischemia and reperfusion, pantoprazole+cardiac ischemia and reperfusion, and pantoprazole+cardiac reperfusion groups.

Photomicrographs of cardiac tissue from SHAM, PANT+CIR, and PANT+CR. SHAM group: no necrosis, striated cells with well-centralized nuclei of normal color and size. SS+CIR group: Intense coagulation necrosis, no tissue loss, with mild myocytolysis (local loss of myocardial syncytium), mild vacuolation, and moderate swelling, with moderate pyknosis (decentralized nuclei and chromatin condensation) and/or karyolysis (nuclear loss) (Figure 3). PANT+CIR group: Intense coagulation necrosis, with intense tissue loss, with areas of myocytolysis, muscle fibers with swelling (increased cell volume due to water accumulation due to reversible ionic imbalance), intense vacuolation, and cells in pyknosis or karyolysis. PANT+CR group: no tissue loss, with moderate pyknosis, with mild karyolysis (absence of nucleus and cytoplasmic eosinophilia) and moderate swelling, with areas of reversible lesion (granulation tissue) (H&E—with 400× magnification) (Figure 3).

## DISCUSSION

Ghebremariam et al.^7^ demonstrated that PPIs inhibit the enzyme dimethylarginine dimethylaminohydrolase (DDAH), which is responsible for ADMA degradation, causing an increase in this substance. This is why PPIs promote increased ADMA levels in cultured human endothelial cells and, concomitantly, decreased NO production. Similarly, human saphenous vein segments produced less NO in vivo when treated with omeprazole. Furthermore, it was demonstrated that mice treated with subcutaneous injections of lansoprazole showed a 20% increase in serum ADMA levels.

Indeed, studies have shown that in both healthy individuals and patients with CVD, an increase in plasma ADMA concentration is associated with an increased risk of MACE and mortality. Furthermore, patients with peripheral or coronary artery disease (CAD) who have elevated plasma ADMA levels are more likely to experience MACE and sudden death. Longitudinal community-based studies have shown that increased plasma ADMA concentrations can be considered a risk factor for the general population^10-12^.

According to Ghebremariam et al., MACE can increase by 30% over a 24-year period, with a 20–30% increase in plasma ADMA. Similar results were observed in the Framingham Offspring Study^7^. Thus, if a patient's "ADHA reserve" is lost as a result of vascular oxidative stress or metabolic disturbances, the PPI-induced increase may be even greater in that patient^10-12^.

Several metabolic disorders promote increased endothelial oxidative stress and, consequently, decreased endothelial ADMA activity. Among them, we can highlight hyperlipidemia, hyperglycemia, and hyperhomocysteinemia. These, in turn, decrease the degradation and increase endothelial and systemic ADMA levels^20^. This produces a considerable impairment of endothelial NOS enzyme activity and, consequently, of NO production, causing dysregulation of vasomotor tone and increased oxidative stress. Furthermore, studies evaluating coronary and brachial artery vasoreactivity have shown that impaired endothelial NOS activity increases the incidence of MACE due to the anti-inflammatory and antiplatelet effects of NO.

There is growing evidence of acquired impairment of DDAH activity through heredity, which would explain why Finnish men with a functional MACE of the DDAH1 gene had elevated plasma levels of ADMA and, therefore, an approximate 50-fold increased risk of CAD and a fivefold increased prevalence of hypertension when compared with non-carrier men in the Kuopio Ischemic Heart Disease Risk Factor Study^21^.

By reducing bradykinin-induced NO production and endothelial NOS, the PPIs can also increase vasoconstriction and decrease vasodilation, which can lead to hypertension. Omeprazole also inhibits the production of metabolites of prostaglandin I_2_, specifically 6-keto-prostaglandin 1α^22^. Araújo et al.^23^ showed that in a rat model, omeprazole, just before myocardial Ischemia and reperfusion, raised blood levels of cardiac damage indicators and the incidence of atrioventricular block with 100% mortality. H^+^/K^+^-ATPase messenger ribonucleic acid and protein were shown to be expressed in human and rabbit cardiac tissue, and the myocardium's physiological and biochemical functions were proven by recently published data. Moreover, H^+^/K^+^-ATPase may be crucial for maintaining cardiac K^+^ and H^+^ homeostasis in rat hearts, according to Schillinger et al.^24^. Therefore, inhibition of H^+^/K^+^-ATPase may result in cellular acidosis, which is known to decrease cardiac contractility primarily at the level of myofilament response to intracellular [Ca^2+^]^24,25^.

Thus, a more plausible explanation for the relationship between MACE and mortality could be a PPI-induced degradation of DDAH activity and the dysregulation of vascular NOS that follows. The FDA may become more concerned after reading the new data on a potential link between PPIs and MAC^10-12^.

We believe that the data obtained in this study are relevant and possibly impactful in relation to the impacts caused by PANT when administered before CR, however, we know that further experimental studies are needed so that the cardioprotection observed in our study can be suggested and implemented in the pharmacotherapy of human patients.

## CONCLUSION

Our results indicate that when PANT is administered before CIR, it promotes cardiotoxicity; however, when administered before CR, it promotes cardioprotection in Wistar rats.

## Data Availability

The datasets generated and/or analyzed during the current study are available from the corresponding author upon reasonable request.
